# Porous Chitosan/Hydroxyapatite Composite Microspheres for Vancomycin Loading and Releasing

**DOI:** 10.3390/pharmaceutics16060730

**Published:** 2024-05-29

**Authors:** Meng-Ying Wu, Yi-Ting Kuo, I-Fang Kao, Shiow-Kang Yen

**Affiliations:** 1 Department of Materials Science and Engineering, National Chung Hsing University, Taichung 402, Taiwan; slimu16882013@gmail.com (M.-Y.W.); firegirl1215@hotmail.com (Y.-T.K.);; 2 Department of Orthopedics, National Defense Medical Center, Taipei 114, Taiwan; 3 Department of Orthopedics, Taichung Armed Forces General Hospital, Taichung 404, Taiwan

**Keywords:** porous chitosan/hydroxyapatite microspheres, vancomycin, drug loading and releasing, inhibiting zone

## Abstract

Porous chitosan/hydroxyapatite (Chi-HAp) composite microspheres were prepared in an aqueous solution containing chitosan, calcium nitrate, and ammonium dihydrogen phosphate by using a hydrothermal method at various temperatures. The investigation indicated that temperature significantly impacted the final product’s appearance. Hydroxyapatite (HAp) coupled with dicalcium phosphate dihydrate (DCPD) flakes were obviously found at 65 and 70 °C, while the latter gradually disappeared at higher temperatures. Conversely, synthesis at 90 °C led to smaller particle sizes due to the broken chitosan chains. The microspheres synthesized at 75 °C were selected for further analysis, revealing porous structures with specific surface areas of 36.66 m^2^/g, pores ranging from 3 to 100 nm, and pore volumes of 0.58 cm^3^/g. Vancomycin (VCM), an antibiotic, was then absorbed on and released from the microspheres derived at 75 °C, with a drug entrapment efficiency of 20% and a release duration exceeding 20 days. The bacteriostatic activity of the VCM/composite microspheres against *Staphylococcus aureus* increased with the VCM concentration and immersion time, revealing a stable inhibition zone diameter of approximately 4.3 mm from 24 to 96 h, and this indicated the retained stability and efficacy of the VCM during the encapsulating process.

## 1. Introduction

Bone infections, particularly osteomyelitis, pose significant challenges in healthcare. Current treatment methods often involve the surgical removal of necrotic bone tissue and repeated irrigations, accompanied by the prolonged administration of high systemic doses of antibiotics, leading to undesirable side effects [1,2,3]. To address this issue, researchers have explored drug delivery systems that facilitate controlled antibiotic release for localized therapy. The development of a biodegradable and osteoconductive drug delivery system holds promise as it could eliminate the necessity for additional surgeries to remove a drug carrier, offering a more effective solution to combat bone infections [4,5].

Calcium phosphates (CaP) have garnered significant attention due to their unique physiochemical and biological characteristics. Hydroxyapatite (HAp, Ca_10_(PO_4_)_6_(OH)_2_) stands out as one of the most extensively studied variants, prized for its biocompatibility, non-toxicity, resorb ability, and osteo-conductivity [6,7]. Bearing a structure akin to bone minerals, HAp can integrate with bone tissue, fostering direct bonding [8,9]. Various fabrication methods exist for HAp production, including hydrothermal methods [10,11,12], solid-state reactions [13,14], sol-gel synthesis [15,16], precipitation [17,18], microemulsion synthesis [19,20], and microwave synthesis [21,22].

**Figure 1:**
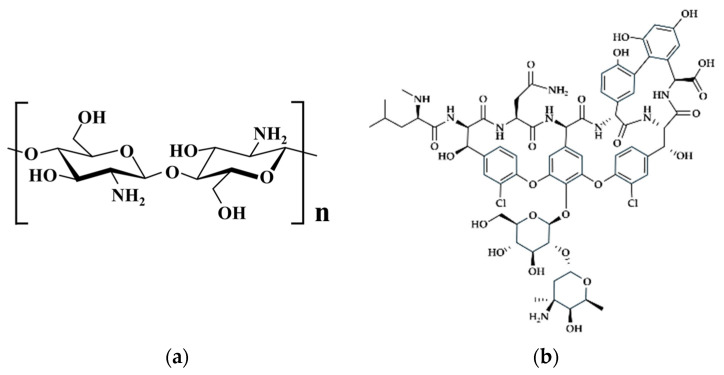
(**a**) Chitosan structure and (**b**) vancomycin structure.

Chitosan, a naturally occurring polymer, exhibits a myriad of advantageous properties, including hydrophilicity, biocompatibility, bio-adhesion abilities, and biodegradability, with minimal toxicity [23,24,25,26]. Its versatility in drug delivery systems is exemplified by its utilization in microsphere formulations [27,28,29]. Various techniques can be employed to fabricate chitosan microspheres, such as spray drying [30,31], emulsification/solvent evaporation [32,33], ionotropic gelation [34,35], and coacervation [36,37]. Derived from the deacetylation of chitin, chitosan consists of 2-amino-2-deoxy-D-glucose and 2-acetamido-2-deoxy-D-glucose units [38,39], making it one of the richest natural polysaccharides full of amino and hydroxyl bonds. The structural representation of chitosan is depicted in Figure 1a.

The combination of chitosan and HAp has emerged as a promising material for bone tissue scaffolds [19,40,41]. This composite material has been utilized as a drug delivery system for proteins, as well as for the treatment of bone infections and defects [27,42].

Vancomycin (VCM), depicted in Figure 1b, is a glycopeptide antibiotic frequently used in the treatment of osteomyelitis [43]. It demonstrates effectiveness against nearly all strains of *Staphylococcus aureus*, with only a few anti-methicillin coagulase-negative *Staphylococcus* strains exhibiting resistance. However, the oral absorption of VCM is challenging, intramuscular injection can be painful, and it is primarily suited for intravenous administration in systemic infections [44]. Nevertheless, high doses of vancomycin may lead to adverse effects including ototoxicity, nephrotoxicity, phlebitis, and neutropenia [45].

A previous experiment successfully synthesized HAp composite microspheres with gelatin as a chelator using a hydrothermal method [46,47]. In this study, the gelatin was replaced by chitosan, and various temperatures for the chitosan/CaP composite synthesis were tested to assess their impacts on the structures of the composite microsphere and the phases of the CaP. The chitosan/HAp composite microspheres with the more uniform size distributions were selected, and they underwent characterization using scanning electron microscopy (SEM), X-ray diffraction (XRD), Fourier-transform infrared spectroscopy (FTIR), thermogravimetric analysis (TGA), an energy dispersive spectrometer (EDS), specific surface area analysis, a porosimeter, and chemisorption analyzers. Determinations of the VCM-loaded microspheres and their antibacterial efficacy were conducted to assess their potential as a high-drug-loading and long-term delivery system.

## 2. Materials and Methods

### 2.1. Materials

Calcium nitrate (Ca(NO_3_)_2_·4H_2_O, 98.5%) and ammonium dihydrogen phosphate (NH_4_H_2_PO_4_, 99.0%) were bought from SHOWA (Tokyo, Japan), and we also procured chitosan powder (Fluka Chemical, Buchs, Germany) with a drying loss of ≤10%. We dissolved 3.5 g of chitosan in 300 mL of a 0.33% acetic acid aqueous solution to form a 1.17 wt.% chitosan solution, and this was stirred for one day.

### 2.2. Synthesis of the Porous Chitosan/Hydroxyapatite Composite Microspheres

The chitosan/HAp (Chi-HAp) composite microspheres were synthesized via the hydrothermal method. NH_4_H_2_PO_4_ and Ca(NO_3_)_2_∙4H_2_O, served as the phosphate and calcium sources for HAp, respectively. These compounds were dissolved separately in deionized water (DIW) for containing calcium and phosphate then introduced into an aqueous solution with 1.17 wt.% chitosan while being continuously stirred by a mechanical mixer to derive Ca/P ratio 1.67 (representing the stoichiometric ratio of HAp). The solution was heated and kept at 65, 70, 75, 80, 85, and 90 °C, respectively in a water sink for 30 min until precipitation occurred. The collected precipitate by centrifugation was washed with DIW. Porous Chi-HAp composite microspheres were obtained through filtration and drying processes. The synthesized Chi/HAp powder will be compared with a reference, reagent-grade HAp powder (Sigma-Aldrich, St. Louis, MO, USA), through various material characterizations.

### 2.3. Characterization of the Chi-HAp

#### 2.3.1. Morphology Observations

The dried powders were subjected to gold-coating for morphology observations using scanning electron microscopy (SEM, JSM-5400, JEOL, Tokyo, Japan) and field emission scanning electron microscopy (FE-SEM, JSM-6700F, JEOL, Tokyo, Japan), coupled with energy dispersive spectrometry (EDS, Oxford Inca Energy 400, Oxfordshire, UK) for analyzing the element contents.

#### 2.3.2. X-ray Diffraction (XRD)

The crystal structures were characterized by XRD analysis using an MO3x-HF diffractometer (Mac Science, Yokohama, Japan). The measurements utilized Cu Kα radiation (λ = 1.5418 Å) at voltage of 40 kV and a current of 30 mA, 2θ from 10° to 70° at a scanning rate of 2°/min. Diffraction patterns were generated and referred to the database from the International Centre for Diffraction Data (ICDD, Newton Square, PA, USA) for identification and analysis.

#### 2.3.3. Functional Group Identification

Fourier-transform infrared spectroscopy (FTIR) analysis was conducted using a Bomem DA spectrometer (Toronto, ON, Canada) for the functional group identification. The Chi-HAp was blended with KBr at ratio of 1:100, covering a wavenumber range from 500 to 4000 cm^−1^ to identify the characteristic bonds.

#### 2.3.4. Specific Surface Area (SSA) and Porosity

The SSA of the Chi-HAp was evaluated using nitrogen adsorption with a Micromeritics ASAP 2010 nitrogen adsorption instrument. The multipoint Brunauer–Emmett–Teller (BET) method was employed to determine the SSA, utilizing adsorption data within a relative pressure (P/Po) range of 0.02–0.45. The Barrett–Joyner–Halenda (BJH) method, which is used to determine pore volumes and area distributions in porous substances based on nitrogen isotherms, incorporates a theory proposed by Wheeler that combines BET multilayer adsorption and capillary condensation viewpoints [48]. The nitrogen adsorption volume at a relative pressure (P/Po) of 0.972 was measured to estimate the average pore size and volume.

#### 2.3.5. Thermal Analysis

Thermogravimetric analysis (TGA) was performed at a heating rate of 10 °C/min in ambient air, with measurements reaching up to 800 °C. The recorded weight variations during the analysis helped elucidate the weight loss phenomena as the temperature increased. Differential scanning calorimetry (DSC) was conducted using a Netzsch HT-DSC 404 apparatus (Selb, Germany) at the same heating rate and atmospheric conditions. The DSC provided insights into the endothermic and exothermic reactions occurring within the sample while also enabling estimation of the enthalpy changes.

### 2.4. Drug Loading and Releasing Kinetics

#### 2.4.1. Loading the VCM on the Chi-HAp and the VCM Content Determination

**Figure 2:**
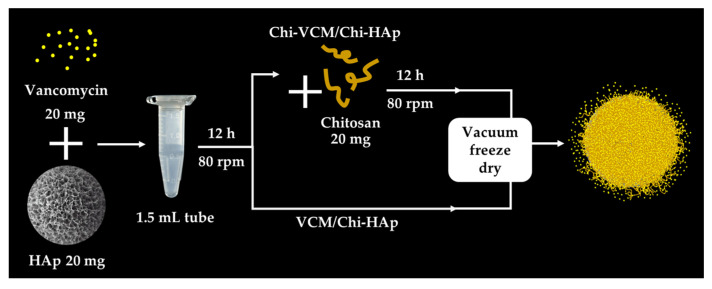
An illustration of the loading process.

VCM, a crucial antibiotic in the treatment of bacterial bone diseases, was incorporated into the composite microspheres. In the VCM loading experiment, one group of 80 mg of Chi-HAp composite microspheres and 20 mg of VCM were mixed in 1 mL of deionized water at 37 °C, blended at 80 rpm for 12 h, and then dried in a 37 °C oven and assigned to VCM/Chi-HAp. In the other group, an extra 20 mg of chitosan was added after 12 h of mixing, as for the previous group, and then the samples were stirred for another 12 h before being dried and assigned to Chi-VCM/Chi-HAp, which was used to demonstrate the role of the chitosan coating. The detailed quantities of VCM, Chi-HAp composite microspheres, and chitosan coating in the VCM/Chi-Hap and the Chi-VCM/Chi-HAp are respectively listed in Table 1. The VCM/Chi-HAp and Chi-VCM/Chi-HAp microspheres were separated via centrifugation and air-dried at room temperature for 48 h. The drug loading process is illustrated in Figure 2.

#### 2.4.2. In Vitro Release of VCM from the VCM/Chi-HAp

An in vitro drug release study was conducted by suspending 20 mg of the VCM/Chi-HAp and Chi-VCM/Chi-HAp in 100 mL of a phosphate-buffered solution (PBS, pH 7.4, Gibco, New York, NY, USA) in vials. A PBS solution with a pH of 7.4 is commonly utilized to assess the in vitro performance of a drug delivery system [49]. Additionally, the average pH of the human body is 7.4 [50]. Drug carriers can be influenced by the composition of a dissolution medium buffer, which can impact drug release kinetics and correlations between the in vitro and in vivo results [51]. The vials were then placed in a sink set at 37 °C and agitated at 80 rpm. After predefined time intervals, samples of 1 mL were collected from the released medium. The withdrawn samples were promptly substituted by an equivalent volume of fresh PBS. The antibiotic contents in the supernatants of the collected samples were quantitatively measured using an ultraviolet–visible (UV/VIS) spectrometer (Hitachi U-3010, Tokyo, Japan), with the VCM analysis conducted at a wavelength of 280 nm, employing a sensitive detection limit of 0.56 µg/mL. A calibration curve was generated by measuring the absorbance of the VCM concentrations ranging from 1 to 500 ppm using UV/VIS spectrophotometry. Measurements were used to calculate the cumulative drug release using the formula shown below (Mc, corrected mass at time *t*; M*t*, apparent mass at time *t*; *v*, volume of the sample taken; and *V*, total volume of the release solution). All experiments were conducted in triplicate.

(1) $$Mc=Mt+\frac{v}{V}\sum_{0}^{t-1}Mt$$

### 2.5. Antibacterial Assessment

The antibacterial assays were conducted using *Staphylococcus aureus*, a prevalent pathogen responsible for severe bone infections. The agar diffusion method [52] was employed to evaluate the antibacterial efficacy of the released antibiotics. *S. aureus* ATCC 6538P was cultured in a nutrient broth comprising 10 g/L of peptone, 2 g/L of beef extract, and 5 g/L of sodium chloride at 37 °C, with agitation for 18 h, resulting in a concentration of 10^7^ colony-forming units (CFU)/mL. We added 1.5 mL of the bacterial suspension to 250 mL of nutrient agar medium, cooled the mixture to approximately 45 °C, and poured it into petri dishes. Next, 70 µL of the extracted medium where antibiotics were released by Chi-VCM/Chi-HAp was dispensed into a central well on an agar plate and incubated at 37 °C for 18 h. The zones of bacterial inhibition were measured and recorded as the average diameter in millimetres, starting from the edge of each well. A negative control group (a solution incubated with microspheres devoid of antibiotics) underwent the same procedure to ensure an accurate comparison.

## 3. Results

### 3.1. Fabrication of the Porous Chi-HAp Powders via a Hydrothermal Method

**Figure 3:**
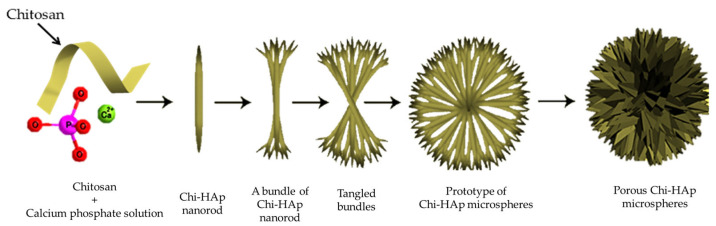
Supposed formation mechanism of the porous chitosan-hydroxyapatite (Chi-HAp) microspheres.

Calcium and phosphate ions were sourced from solutions of Ca(NO_3_)_2_ and NH_4_H_2_PO_4_, respectively, then blended with the chitosan solution. The resultant mixture underwent stirring for 2 min, yielding the composite Chi-Hap powder. The molar ratio of Ca(NO_3_)_2_ to NH_4_H_2_PO_4_ was maintained at 5:3, reflecting the stoichiometric ratio of HAp. Following the drying process, a white powder was collected and subjected to scanning electron microscopy (SEM) analysis to investigate the morphology of the Chi-Hap composite microspheres. Figure 3 illustrates the formation mechanism of the Chi-HAp. In the synthesis process of the Chi-HAp, the chitosan, Ca^2+^, and PO_4_^3−^ first formed Chi-HAp rods, then the bundled rods tangled together to finally form the porous and spherical Chi-HAp microspheres.

#### The Influence of Temperature on the Synthesis Process of the Chi-HAp Composite Microspheres

**Figure 4:**
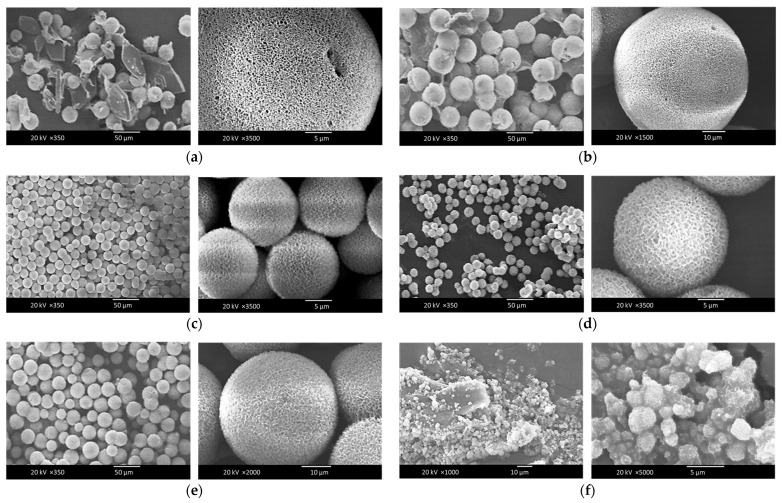
SEM micrographs of the Chi-HAp microspheres synthesized at (**a**) 65 °C, (**b**) 70 °C, (**c**) 75 °C, (**d**) 80 °C, (**e**) 85 °C, and (**f**) 90 °C.

**Figure 5:**
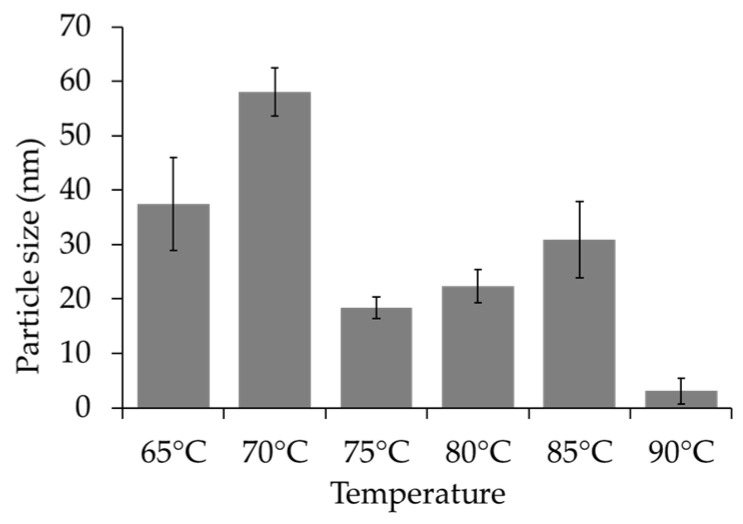
Particle sizes of the Chi-HAp synthesized at 65 °C, 70 °C, 75 °C, 80 °C, 85 °C, and 90 °C.

The SEM images depicting the surface morphology of the composite microspheres at temperatures of 65 °C, 70 °C, 75 °C, 80 °C, 85 °C, and 90 °C are presented in Figure 4. The observations revealed that the powders comprised a combination of spherical and plate-like structures at 65 °C (Figure 4a), where the former corresponded to HAp and the latter to dicalcium phosphate dihydrate (DCPD). The proportion of plate-like particles decreased with increasing temperature, as evidenced by the images in Figure 4b (70 °C) and 4c (75 °C). Plate-like structures were scarcely observed at higher temperatures, such as 80 °C and 85 °C (Figure 4d and Figure 4e, respectively), indicating that elevated temperatures favored the grain growth of HAp. Fewer Ca^2+^ and HPO_4_^2−^ ions were retained in the solution, resulting in reduced DCPD formation. However, increasing the temperature to 90 °C led to the breakdown of the chitosan chains into shorter fragments, thereby yielding smaller Chi-HAp microspheres, as observed in Figure 4f. From Figure 4a,b and Figure 5, it can be observed that the microsphere sizes were more uniform and almost devoid of DCPD when synthesized at 75, 80, and 85 °C. Among these, the samples synthesized at 75 °C revealed the most uniformity in size, and they were selected for further analysis.

### 3.2. Characterization of the Surface Morphologies and Compositions of the Chi-HAp Composite Microspheres through FE-SEM and EDS Analyses

**Figure 6:**
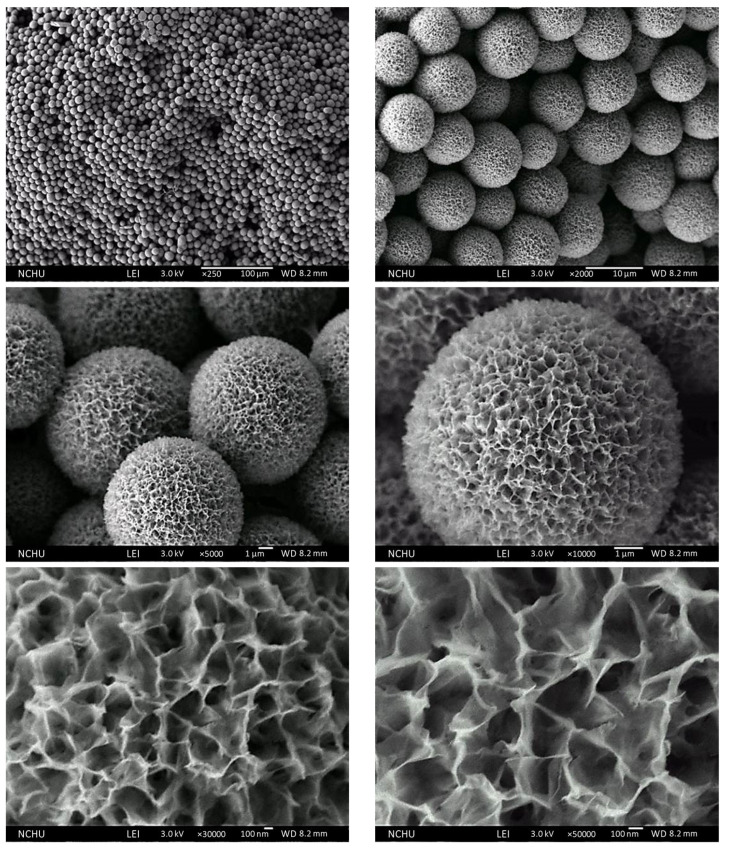
FE-SEM micrographs of the Chi-HAp with a synthesis temperature of 75 °C.

The micrographs in Figure 6 showed that the Chi-HAp composite particles were composed of hydrangea-like flakes, revealing spherical outlooks and more uniform size distributions at approximately 10 µm.

**Figure 7:**
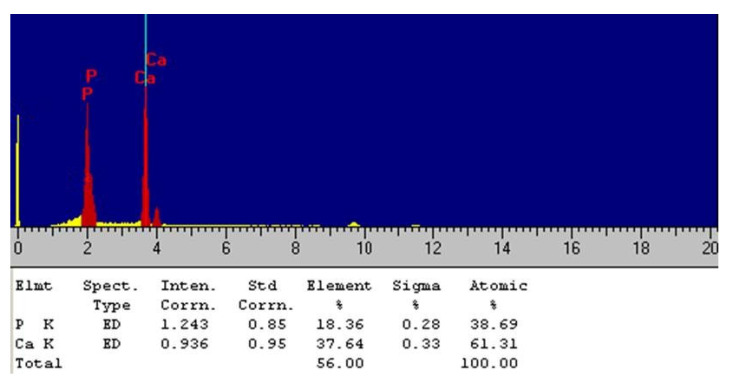
EDS spectrum of the Chi-HAp microspheres.

EDS was employed to determine the calcium-to-phosphorus (Ca/P) ratio of the Chi-HAp composite microspheres. As illustrated in Figure 7,the Kα peak (the left one) was clearly separated from the Kβ one (the right one) for Ca, while they were overlapped for P, and the yellow one was the noise of the background. The EDS spectrum indicated a Ca/P ratio of 1.58 for the Chi-HAp composite microspheres, which was less than the Ca/P ratio of pure HAp (1.67), also called calcium-deficient HAp, while higher than that prepared at 65 °C (1.43), as indicated in a previous report [53].

### 3.3. Crystal Structures

**Figure 8:**
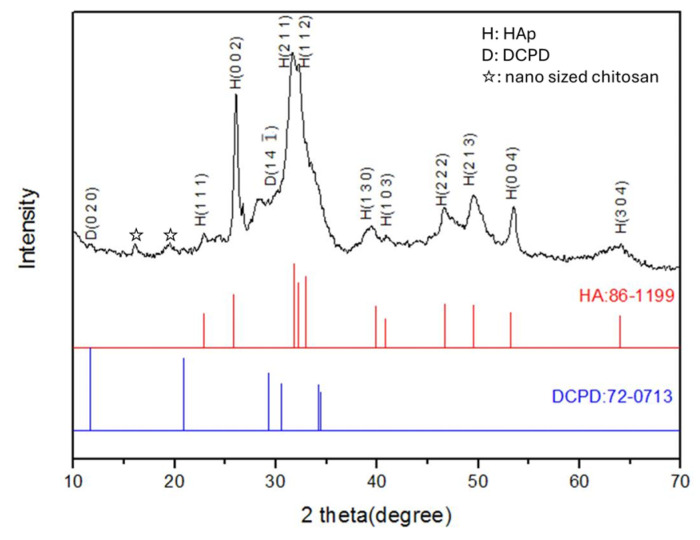
XRD pattern of the Chi-HAp compared with ICDD file no. 86-1199 and no. 72-0713.

The crystal structures of the Chi-HAp composite powders are illustrated in Figure 8. The diffraction patterns were examined and compared to the ICDD database, revealing a close alignment with peaks corresponding to ICDD card numbers 86-1199 and 72-0713 for HAp and DCPD, respectively. The diffraction peaks associated with the crystal planes of the HAp were observed at 22.873° (111), 25.883° (002), 31.792° (211), 32.206° (112), 39.830° (130), 40.848° (103), 46.726° (222), 49.508° (213), 53.220° (004), and 64.018° (304). Weak diffraction peaks corresponding to DCPD were observed at 11.650° (020) and 29.296° (141), and nano-sized type Ⅱ chitosan [54] was also detected, which was indicative of a lesser crystalline phase and/or smaller crystal size for the DCPD. The composite microspheres prepared at 65 °C revealed a higher peak intensity for the DCPD, as indicated in a previous report [53]. Given the superior osteo-conductivity of the HAp compared to the DCPD and that its calcium to phosphorus ratio was akin to that of human bone, it emerged as a highly promising biomaterial for biomedical applications.

### 3.4. SSA and Porosity of the Chi-HAp

**Figure 9:**
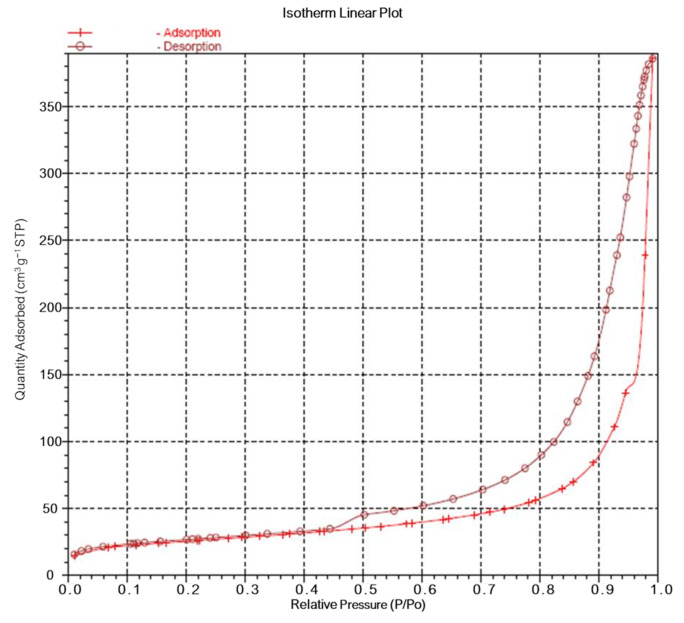
Nitrogen adsorption/desorption isotherm derived from the BET method.

The nitrogen adsorption/desorption isotherm derived from the BET method is presented in Figure 9. The observed hysteresis loop conformed to the H3 type, which is typically associated with porous structures assembled by micro-flakes [55]. This classification system categorizes hysteresis loops into four distinct types, denoted as H1, H2, H3, and H4, each indicative of different structural characteristics. While an H1-type hysteresis is attributed to agglomerates or uniform spheres comprising a porous material, the definition of the H2 type remains less clearly defined due to various potential contributing factors. On the other hand, an H4-type hysteresis is primarily associated with narrow aperture structures. The resemblance of the hysteresis loop observed in the prepared microspheres to the H3 type suggests a porous structure assembled by micro-flakes, consistent with the visual examination of the composite material, as shown in Figure 6.

**Figure 10:**
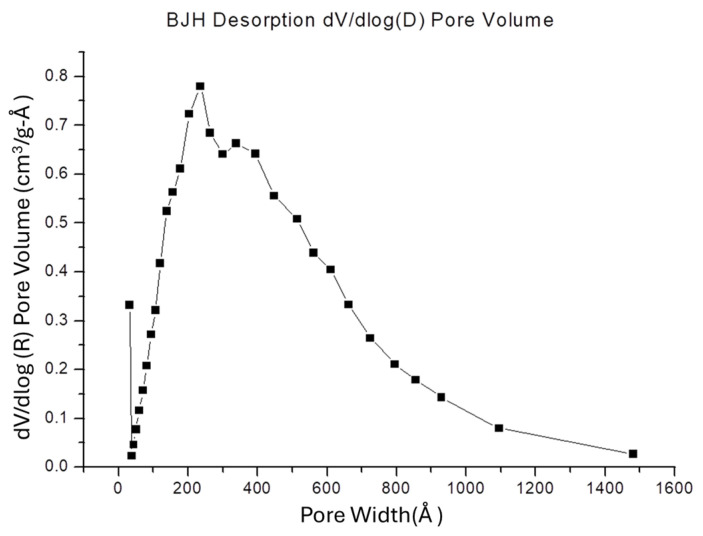
Distribution of the BJH desorption pore sizes.

Based on the BET analysis, the surface areas of the Chi-HAp composite microspheres were determined to be 36.66 m^2^/g (Table 2). Employing the BJH adsorption and desorption method, it was found that the SSAs were within the range of 17 to 3000 Å in width, yielding SSA values of 85.52 m^2^/g and 140.99 m^2^/g, respectively. The pore volume for the BJH adsorption was calculated as 0.5874 cm^3^/g while that for desorption was 0.5953 cm^3^/g. The analysis of the BJH desorption pore size distribution, illustrated in Figure 10, revealed the presence of peaks at 30, 200, and 400 Å, indicating high surface areas and significant porosity. These findings underscored the potential of the Chi-HAp composite microspheres as an effective drug carrier system.

The SEM observations also confirmed the morphologies of the microspheres to be comprised of petal-like flakes, characteristics that were consistent with the BET analysis classification (H3 type). This correspondence between the SEM observations and the BET analysis enhanced the reliability of the structural characterization of the composite material.

### 3.5. Thermal Analysis

**Figure 11:**
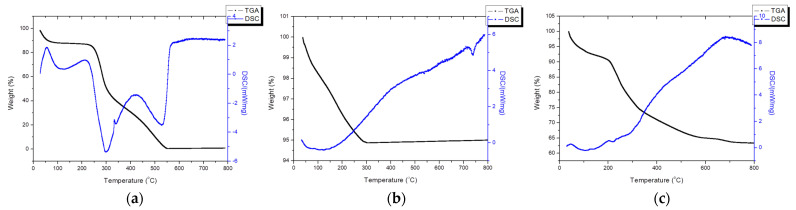
TGA/DSC graphics of (**a**) chitosan, (**b**) commercial HAp, and (**c**) Chi-HAp in air.

The TGA/DSC diagram of chitosan conducted under atmospheric conditions is presented in Figure 11a, revealing a distinct pattern of weight loss characterized by three distinct steps. The initial step, accounting for approximately 12% of the weight loss, corresponded to the evaporation of water molecules, as evidenced by an endothermic peak observed from room temperature to 100 °C. The second step, resulting in approximately 43% of the weight loss, was derived from the condensation of the hydroxyl and amino bonds in the chitosan, as indicated by an exothermic peak at approximately 300 °C. The final step involved the oxidation of the retained carbon in the presence of air, as characterized by another exothermic region initiating from 420 °C. Chitosan undergoes complete combustion at 550 °C, which thereby concluded the thermal degradation process.

The TGA/DSC diagram for the commercial HAp is depicted in Figure 11b. The predominant weight loss observed was approximately 5%, occurring gradually from room temperature to 300 °C due to the desorption of adsorbed water molecules within the HAp structure. From 300 °C to 800 °C, there were no discernible endothermic or exothermic peaks and no obvious weight loss. However, a small endothermic peak was observed at approximately 725 °C, possibly due to the crystal growth of the HAp.

The TGA/DSC diagram for the Chi-HAp composite microspheres is shown in Figure 11c. From room temperature to 225 °C, a weight loss of 9% was found, resulting from the evaporation of water molecules originally adsorbed on the microspheres’ surfaces. A weight loss of 17% was more evident from 225 °C to 350 °C, attributed to the condensation of the hydroxyl and amino bonds in the chitosan within the Chi-HAp. A weight loss of 10% slowly proceeded from 350 °C to 700 °C due to the combustion of carbon. Unlike the pure chitosan, no obvious endothermic or exothermic peaks were observed in the Chi-HAp composite since the evaporation of the adsorbed water was mixed with the condensation of the hydroxyl and amino bonds in the chitosan. The final retained weight of 64% was dominated by HAp; in other words, the composite microspheres were comprised of 64% HAp, 9% physically adsorbed water, and 27% chitosan.

### 3.6. Functional Group Identification through an FTIR Spectrograph Spectrometer

**Figure 12:**
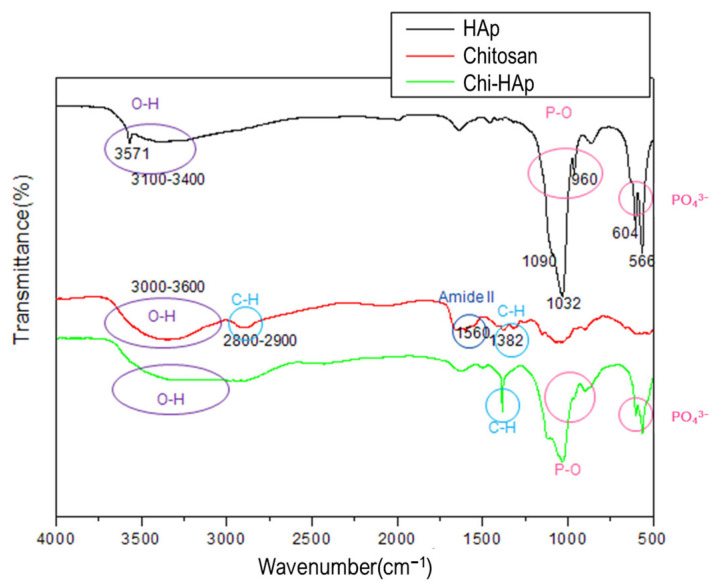
FTIR spectra acquired from the commercial HAp, chitosan, and Chi-HAp, respectively.

The functional group identification of the commercial HAp (shown in black), chitosan (shown in red), and Chi-HAp composite microspheres (shown in green) is presented in Figure 12. In the first curve representing the FTIR spectrum of the commercial HAp, characteristic absorption bands were observed. The hydroxyl (OH) groups were identified at wavenumbers ranging from 3400 to 3100 cm^−1^, with a prominent peak at 3571 cm^−1^. The phosphate absorption bands were apparent at 1090, 1032, 960, 604, and 566 cm^−1^, attributed to the stretching vibrations of the P-O or PO_4_^3−^ bonds. These observed absorption bands aligned with the characteristic features of a typical HAp FTIR spectrum.

The presence of hydroxyl groups was evidenced by a broad band spanning from 3000 to 3600 cm^−1^. The C-H bonds observed at 2800–2900 cm^−1^ and 1382 cm^−1^, the presence of an amine bond at wavenumber 1560 cm^−1^, the N-H bonds at 1318 and 1254 cm^−1^, and the C-O ether bonds at 1150 and 1032 cm^−1^ were consistent with the known attributions of chitosan, as illustrated by the second curve in Figure 12.

Upon examination of the FTIR spectrum of the prepared Chi-HAp composite microspheres, depicted by the third curve in Figure 12, the chemical bonds of both the HAp and the chitosan were observed. The characteristic peaks corresponding to the HAp were identified at 1032, 960, 604, and 566 cm^−1^, while distinct chitosan peaks were also discernible. The intensity of the C-H stretching peak at 1382 cm^−1^ exhibited a significant increase compared to that of the pure chitosan. The dispersion of the chitosan by the HAp suggested the possibility of the more efficient exposure of the C-H bonds within the composite microspheres. Compared to the other two materials under the same scale, the intensity of the functional group for the pure HAp was stronger, corresponding to the more pronounced peaks.

### 3.7. Drug Release

**Figure 13:**
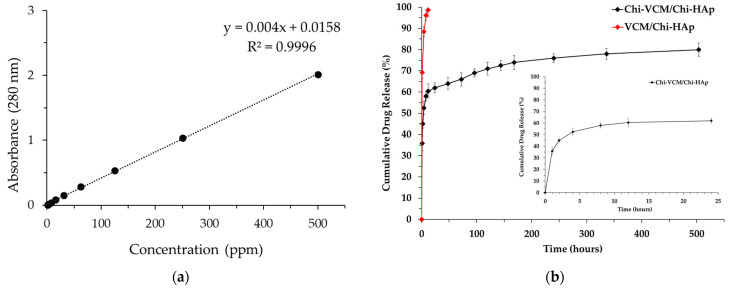
(**a**) The calibration curve of the VCM concentrations. (**b**) The VCM release curves from VCM/Chi-HAp and Chi-VCM/Chi-Hap, respectively, and the right inserted profile is the enlargement of the Chi-VCM/Chi-HAp for the initial 24 h.

The absorbance of the VCM released from the VCM/Chi-HAp in the PBS was measured using UV/VIS spectrophotometry. The values were then applied to the following calibration curve: y = 0.04x + 0.0158, as shown in Figure 13a. The drug release profiles are depicted in Figure 13b, with red demonstrating that in the absence of chitosan, the drug was completely released within a 12 h timeframe, and the right inserted profile is the enlargement of the initial 24 h. Conversely, the introduction of chitosan prolonged the duration of drug release to 21 days. This extended-release period was attributed to the formation of hydrogen-bonding interactions between the chitosan and both vancomycin and HAp. These interactions enhanced the connection between the drug and the carrier, resulting in the development of a more durable composite structure that extended the drug release timeline. This highlighted the significant impact of chitosan on regulating drug release. Three distinct phases occurred during the release process. Initially, a rapid release rate observed within the first 4 h could be attributed to the dispersion of drug molecules on the surfaces of the composite microspheres. From hours 8 to 168 (day 7), a moderate release rate was observed, which could be attributed to the gradual degradation of the chitosan coatings on the composite microspheres, resulting in a linear release curve. Finally, after the first week, a more gradual release rate was observed, likely resulting from the release of the drug molecules residing within the macropores and mesopores of the microspheres.

Compared to the VCM carried solely by chitosan, the initial burst release trend was similar. However, at the same release time (4 h), the release amounts for the VCM-chitosan and the VCM/Chi-HAp were 70% and 52.5%, respectively [56]. Obviously, the initial burst of the latter was less than that of the former. Other carriers for VCM include MgCa_7_Si_4_O_16_, MgCaSi_2_O_6_, and MgCa_2_Si_2_O_7_, with drug-loading capacities of 15.9, 16.6, and 17.3 mg/g, respectively. After being coated with PLGA and subjected to drug release experiments, the cumulative release amounts after 168 h were approximately 88%, 65%, and 75%, respectively [57]. In comparison, the cumulative release amount for the Chi-Hap coated with chitosan was 67% at 168 h, which was similar to that of MgCaSi_2_O_6_. However, Chi-HAp has a drug encapsulation capacity of 250 mg/g, which is significantly higher than the other three carriers. The pores in the Chi-HAp extended the release duration and may support bone integration, which was found in a previous report for porous gelatine/hydroxyapatite composite microspheres [58].

**Figure 14:**
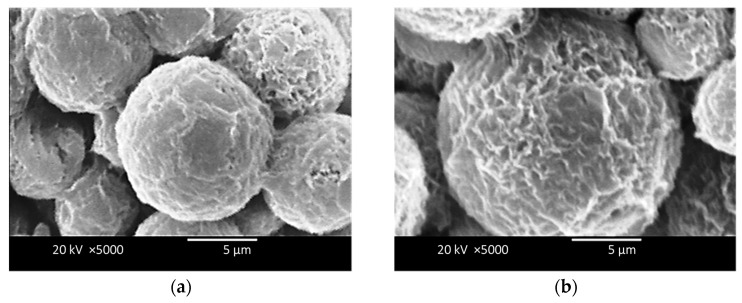

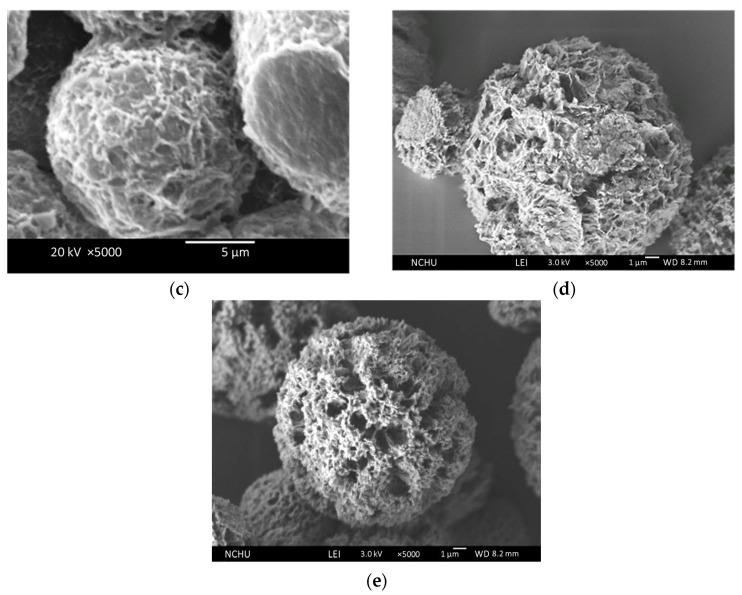
The morphologies of the Chi-HAp after drug release for (**a**) 1 h, (**b**) 8 h, (**c**) 12 h, (**d**) 7 days, and (**e**) 28 days.

**Figure 15:**
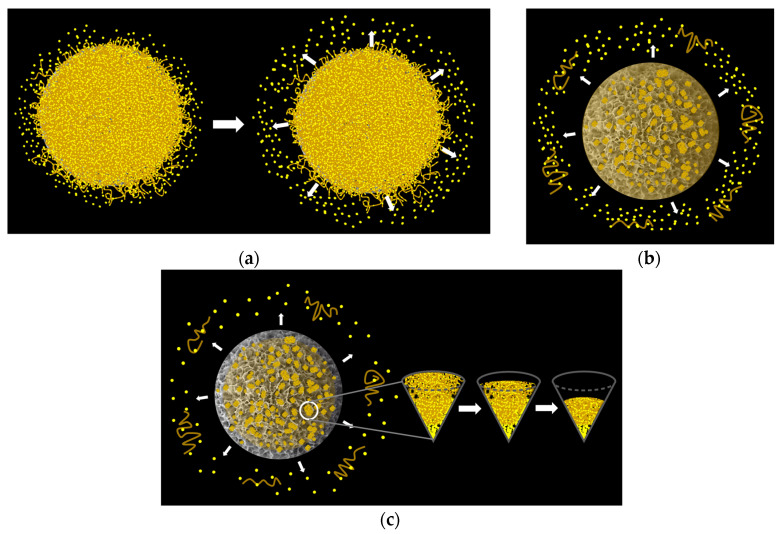
A schematic of the drug-release process involving (**a**) the dissolution of the dried VCM on the surface of a microsphere in 4 h, (**b**) the swelling and degradation of the chitosan on the surface of a microsphere before day 7, and (**c**) the swelling and degradation of the chitosan within the mesopores and/or macropores of a microsphere after day 7. The big arrows indicate the drug release process and the small ones show the releasing directions.

The SEM observations of the Chi-VCM/Chi-HAp microspheres at various timepoints (1 h, 8 h, 12 h, 7 days, and 28 days) are presented in Figure 14a–e. For the early-stage drug release observations, standard SEM was sufficient. However, compared with standard SEM, FE-SEM with more field depth was applied to reveal clearer images of the pores exposed by the microspheres after the chitosan degradation in the later stages. Therefore, the observations from the seventh day onward were conducted using FE-SEM. The drug-release process is illustrated in Figure 15, where light yellow denotes the VCM and khaki denotes the chitosan. The initial smoother surface observed at 1 h indicated minimal degradation of the chitosan coating layer, corresponding to Figure 15a, with the dissolution of the dried VCM on the surface at 4 h. As the release progressed, the surfaces became increasingly crumpled, as shown in Figure 14b,c, which was indicative of the degradation of the chitosan coating layer over time, corresponding to Figure 15b, with the swelling and degradation of the chitosan on the surfaces of microspheres before day 7. The initial presence of macropores greater than 1 µm, as depicted in Figure 14d, suggested the beginning of the chitosan degradation. More pores were found after 28 days, while the geometries of the pores were tuned, possibly due to the remodelling of the HAp by dissolution and precipitation, as presented in Figure 14e, and corresponding to Figure 15c, the swelling and degradation of the chitosan within the mesopores and/or macropores of microspheres was apparent after day 7. The carbon contents of the drug-loaded spheres were decreasing with the release duration, as provided in Table 3, and this was consistent with the argument of chitosan degradation.

### 3.8. Antibacterial Assessment

**Figure 16:**
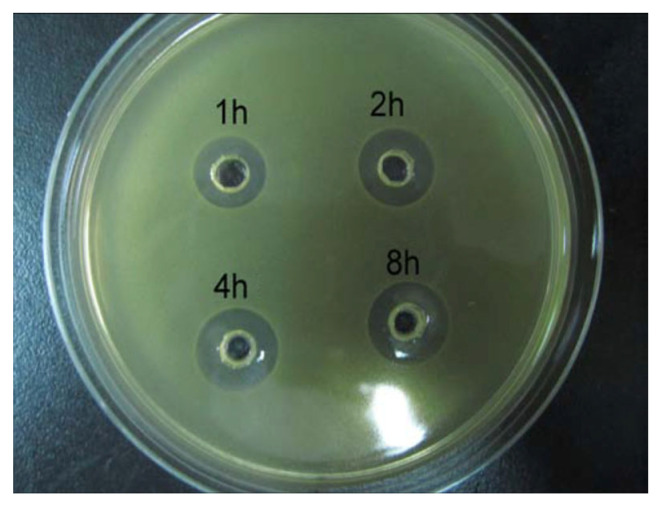
Bacterial inhibition zones around the holes that were initially filled with 70 μL of elution fluids containing the VCM released from the Chi-HAp microspheres.

A presentative inhibiting zone test is shown in Figure 16, with the wells initially loaded with 70 µL of medium containing the VCM released from the microspheres at 1, 2, 4, and 8 h. The presence of a light, circular area indicated an inhibition zone where bacterial proliferation was absent. Some past research has indicated that the effectiveness of chitosan against *S. aureus* demonstrated a significant effect with an inhibition zone of 8 mm within 24 h [59]. However, the samples tested in this experiment were derived from an extracted medium where antibiotics were released by the VCM/Chi-HAp, indicating no effect of chitosan, and no inhibitory effect was observed in the control samples (the solution incubated with microspheres devoid of loaded antibiotics). This suggested that the observed inhibitory effect was solely attributable to the released antibiotics from the microspheres, without any interference of other materials.

**Figure 17:**
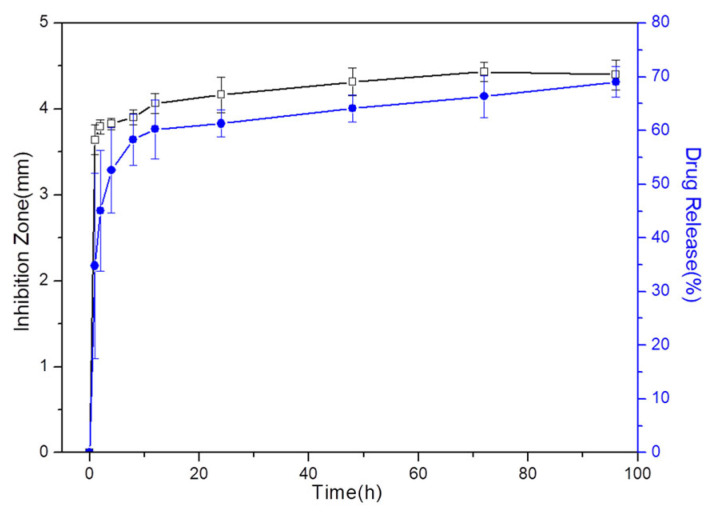
The bacterial inhibition zones of the VCM released from the Chi-VCM/Chi-HAp microspheres into the PBS solution corresponded to the cumulative concentrations.

The results of the bacterial inhibition zones of the VCM released from the microspheres, tested with *Staphylococcus aureus*, are presented in Figure 17. The inhibitory zones initially measured 3.5 mm after 1 h, gradually increasing to approximately 4.3 mm after 8 h. A stable inhibitory effect against bacteria was then achieved, lasting for 4 days. Given the initial burst and sustained release observed in the vancomycin release profile (Figure 13b), the antibacterial effect of the microspheres on the inhibitory zone sizes exhibited a rapid increase stage, followed by a stable stage. Furthermore, the inhibition zones of the VCM decreased with increasing time during the later stage, attributable to the withdrawal of the elution fluids at predetermined intervals and the subsequent replenishment with fresh PBS solution. The observed inhibition zones for the VCM released at various immersion times were directly correlated with the measured concentrations at each stage. This indicated that the antibiotics released from the microspheres were capable of inhibiting most of the bacteria within 100 mL within 1 h, and thus, they hold promise for the treatment of osteomyelitis. These findings underscored the chemical stability and full activity of the VCM encapsulated within the Chi-HAp microspheres following release.

## 4. Conclusions

The chitosan/hydroxyapatite (Chi-HAp) composite microspheres were successfully synthesized via a hydrothermal method, utilizing a broad synthesis temperature range of 75 to 85 °C. The SEM analysis revealed that the obtained Chi-HAp microspheres exhibited spherical morphologies, with uniform sizes ranging from 10 to 20 µm, and they possessed porous structures. The Chi-HAp microspheres exhibited high SSAs of 36.66 m^2^/g and significant pore volumes of 0.58 cm^3^/g. The thermal analysis further indicated that the composite microspheres comprised 64% HAp, 9% physically adsorbed water, and 27% chitosan. These characteristics, including the porous nature, high surface area, and pore volume, suggested the potential for drug encapsulation and delivery.

The release duration of the Chi-VCM/Chi-HAp microspheres was more than 21 days due to the incorporation of chitosan, attributed to its coatings on the microspheres. The in vitro drug release exhibited three distinct phases. The initial phase, within 4 h, revealed an initial burst of drug release, attributed to the dissolution of drug molecules from the surface. From hour 8 to day 7, a moderate release rate was observed, stemming from the dissociation of chitosan and vancomycin coatings on the composite microspheres, resulting in a linear release profile. A gradual and sustained release phase was observed after one week, caused by the release of drug molecules from the macropores and mesopores within the microspheres.

The eluted fluid from the VCM release tests exhibited distinct bacterial inhibitory zones against *Staphylococcus aureus*, with the sizes of the inhibitory zones directly correlated to the measured drug concentrations at each immersion time. After eight hours, a stable inhibitory effect against bacteria was achieved, lasting for four days, with an inhibitory zone diameter of 4 mm. This indicated that VCM loaded into the composite microspheres remained chemically stable and biologically active during the encapsulating process and after release. Obviously, the Chi-HAp composite microspheres developed in this study demonstrate significant potential as drug carriers for the treatment of osteomyelitis.

## Figures and Tables

**Table 1:** Quantities of vancomycin (VCM), chitosan-hydroxyapatite (Chi-HAp) composite microspheres, and chitosan coating in the VCM/Chi-HAp and the Chi-VCM/Chi-HAp.

	VCM (mg)	Chi-HAp (mg)	Chitosan (mg)	VCM:Chi-HAp
Chitosan-coated VCM/Chi-HAp	20	80	20	1:4
VCM/Chi-HAp	20	80	0	1:4

**Table 2:** Specific surface area (SSAs), pore volumes, and pore sizes.

	BET	BJH Adsorption	BJH Desorption
SSA	36.66 ± 1.32 m^2^/g	85.52 ± 2.85 m^2^/g	140.99 ± 4.23 m^2^/g
Pore volume		0.5874 ± 0.0125 cm^3^/g	0.5953 ± 0.0274 cm^3^/g
Pore size	403.5 ± 9.65 Å	271.6 ± 8.12 Å	168.9 ± 5.94 Å

**Table 3:** The content of carbon at different times.

Time	0 h	1 h	2 h	4 h	8 h	1 Day	7 Days	28 Days
Content of C	60.66 ± 1.85%	56.89 ± 2.59%	56.43 ± 1.63%	56.01 ± 1.17%	47.87 ± 1.24%	34.03 ± 1.82%	28.98 ± 1.54%	20.01 ± 2.12%

## Data Availability

Data are contained within the article.
